# The Dual-Active Histamine H_3_ Receptor Antagonist and Acetylcholine Esterase Inhibitor E100 Alleviates Autistic-Like Behaviors and Oxidative Stress in Valproic Acid Induced Autism in Mice

**DOI:** 10.3390/ijms21113996

**Published:** 2020-06-03

**Authors:** Nermin Eissa, Sheikh Azimullah, Petrilla Jayaprakash, Richard L. Jayaraj, David Reiner, Shreesh K. Ojha, Rami Beiram, Holger Stark, Dorota Łażewska, Katarzyna Kieć-Kononowicz, Bassem Sadek

**Affiliations:** 1 Department of Pharmacology & Therapeutics, College of Medicine and Health Sciences, United Arab Emirates University, Al Ain P.O. Box 17666, UAE; 201690014@uaeu.ac.ae (N.E.); azim.sheikh@uaeu.ac.ae (S.A.); petrilla.jp@uaeu.ac.ae (P.J.); richardlj@uaeu.ac.ae (R.L.J.); shreeshojha@uaeu.ac.ae (S.K.O.); rbeiram@uaeu.ac.ae (R.B.); 2 Zayed Center for Health Sciences, United Arab Emirates University, Al Ain P.O. Box 17666, UAE; 3 Institute of Pharmaceutical and Medicinal Chemistry, Heinrich Heine University Düsseldorf, Universitaetsstr. 1, 40225 Düsseldorf, Germany; David.Reiner@uni-duesseldorf.de (D.R.); stark@hhu.de (H.S.); 4 Department of Technology and Biotechnology of Drugs, Faculty of Pharmacy, Jagiellonian University-Medical College, Medyczna 9 St., 30-688 Kraków, Poland; dlazewska@cm-uj.krakow.pl (D.Ł.); mfkonono@cyf-kr.edu.pl (K.K.-K.)

**Keywords:** VPA-induced autism-like behaviors, mice, sociability, repetitive behaviors, anxiety histamine H3R, antagonist, acetylcholine esterase inhibitor, E100, oxidative stress, cerebellum

## Abstract

The histamine H3 receptor (H3R) functions as auto- and hetero-receptors, regulating the release of brain histamine (HA) and acetylcholine (ACh), respectively. The enzyme acetylcholine esterase (AChE) is involved in the metabolism of brain ACh. Both brain HA and ACh are implicated in several cognitive disorders like Alzheimer’s disease, schizophrenia, anxiety, and narcolepsy, all of which are comorbid with autistic spectrum disorder (ASD). Therefore, the novel dual-active ligand E100 with high H3R antagonist affinity (hH3R: *K*_i_ = 203 nM) and balanced AChE inhibitory effect (*Ee*AChE: IC_50_ = 2 µM and *Eq*BuChE: IC_50_ = 2 µM) was investigated on autistic-like sociability, repetitive/compulsive behaviour, anxiety, and oxidative stress in male C57BL/6 mice model of ASD induced by prenatal exposure to valproic acid (VPA, 500 mg/kg, intraperitoneal (i.p.)). Subchronic systemic administration with E100 (5, 10, and 15 mg/kg, i.p.) significantly and dose-dependently attenuated sociability deficits of autistic (VPA) mice in three-chamber behaviour (TCB) test (all *p* < 0.05). Moreover, E100 significantly improved repetitive and compulsive behaviors by reducing the increased percentage of marbles buried in marble-burying behaviour (MBB) (all *p* < 0.05). Furthermore, pre-treatment with E100 (10 and 15 mg/kg, i.p.) corrected decreased anxiety levels (*p* < 0.05), however, failed to restore hyperactivity observed in elevated plus maze (EPM) test. In addition, E100 (10 mg/kg, i.p.) mitigated oxidative stress status by increasing the levels of decreased glutathione (GSH), superoxide dismutase (SOD), and catalase (CAT), and decreasing the elevated levels of malondialdehyde (MDA) in the cerebellar tissues (all *p* < 0.05). Additionally, E100 (10 mg/kg, i.p.) significantly reduced the elevated levels of AChE activity in VPA mice (*p* < 0.05). These results demonstrate the promising effects of E100 on in-vivo VPA-induced ASD-like features in mice, and provide evidence that a potent dual-active H3R antagonist and AChE inhibitor (AChEI) is a potential drug candidate for future therapeutic management of autistic-like behaviours.

## 1. Introduction

Autistic spectrum disorder (ASD) is a neurodevelopmental disorder with a large population prevalence, characterized by impairments in social interaction and restricted/repetitive behavioral pattern or interest [1,2]. Despite its increasing prevalence, the pathophysiology of ASD is still poorly understood [3,4]. The difficulty in understanding the pathophysiology of ASD lies in the complex involvement of several clinical and behavioral symptoms, making clinically accessible specific treatments for ASD often less effective [5,6]. Recent advances in drug developments focus on novel agents with multiple pharmacological effects for multifactorial diseases, such as ASD [7,8,9]. In search of sensitive and specific markers of ASD, numerous research efforts have focused on the study of various brain neurotransmitters [4]. Hence, assessment of the function of numerous brain neurotransmitters, e.g., histamine (HA), acetylcholine (ACh), serotonin (5-HT), dopamine (DA), γ-aminobutyric acid (GABA), and glutamate (Glu) in initial brain growth encourages to be an important area of research in the field of developing newer therapeutics [4,10,11,12,13,14,15,16,17,18,19,20]. Accordingly, the brain cholinergic neurotransmitter system with ACh has an essential role in controlling ASD-related behavioral features including attention [19], cognitive flexibility [20], social interaction [21], and stereotypical behaviors [9,16,18,22]. Preclinical as well as clinical evidences reveal the involvement of cholinergic system dysfunction in the phenotypic outcomes of ASD-related behavioral features, in both humans and animal models [23]. In ASD patients, there are significant irregularities in the brain cholinergic system. Anatomically there is abnormality in the number and structure of neurons in a basal forebrain cholinergic nucleus of patients diagnosed with ASD [24]. Additionally, a remarkable reduction in the level of choline, a precursor of the neurotransmitter ACh and agonist for nicotinic-cholinergic receptor, was reported in individuals diagnosed with ASD [25]. In addition, abnormalities in the levels of nicotinic ACh receptors were observed in several brain regions, e.g., neocortex, cerebellum, thalamus, and striatum, of patients diagnosed with ASD, with the chief abnormalities being the reduced levels of muscarinic receptors (M1 type) [26,27,28].

Several essential physiological functions e.g., sleep–wake cycle, energy and endocrine homeostasis, sensory and motor functions, cognition, and attention, are controlled by the brain histaminergic system, and as such, are all severely affected in neuropsychiatric disorders [4,29,30,31,32]. Histamine mediates its effects through binding to four known histamine receptor (HR) subtypes belonging to the family of G-protein-coupled receptors, and designated H1 to H4 receptors (H1R–H4R). The histamine H3 receptor (H3R) initially described in 1983 was found to be a constitutively active receptor mostly expressed in the brain and was evaluated pharmacologically to negatively regulate histamine synthesis and release, acting as presynaptic auto-receptors [33,34]. In addition, H3Rs functioning as hetero-receptors can also control the release of other neurotransmitters like ACh, Glu, GABA, 5-HT, and DA in various brain regions [35,36,37,38,39]. Moreover, it has been revealed that H3Rs are predominantly expressed in the central nervous system (CNS), while activation of H1R and H2R mediates slow excitatory postsynaptic potentials. Interestingly, few studies projected the use of HR antagonists in the therapeutic management of autistic behavior. Consequently, famotidine (a histamine H2R antagonist) was projected to be a possible treatment for ASD children [12], since famotidine was revealed to alleviate sociability deficits in a patient with schizophrenia [13], a brain disorder that shares various genetic factors and symptoms with ASD [14,15]. In addition, niaprazine (a histamine H1R antagonist) has weakened features such as unbalanced attention, resistance to alteration, and frustration in patients with ASD [40]. Furthermore, it has been proposed that histamine H3R antagonists are of potential therapeutic future for the treatment of several brain disorders, e.g., Alzheimer’s disease (AD), schizophrenia, and narcolepsy [29,30,31,32]. Accordingly and in an animal model of schizophrenia, H3R antagonist was found to ameliorate behavioral deficiencies, including spatial working memory deficit, an abnormality also found in ASD patients [32]. Besides, antagonism of histamine H3Rs was found to reduce social behavior deficits in rodents exposed to phencyclidine, signifying the promising potential use of H3R antagonist in the therapeutic management of ASD [31,32]. Additionally, ciproxifan, an old-generation H3R antagonist, was found to attenuate impaired sociability and stereotypies in animal model of ASD in which rodents were exposed to valproic acid (VPA) [1].

During pregnancy, environmental risk factors may affect the inflammatory response of new-borns, hence altering postnatal brain development [41]. VPA as an environmental risk factor showed activation in different brain regions, with evidence of long-lasting glia activation in the hippocampus and the cerebellum [42], which are two brain regions associated with autism-related features, namely social interaction and repetitive behaviours [41,43]. Moreover, numerous preclinical experiments indicated that inflammation in the cerebellum may change social behaviours in adult mice, since cerebellum was found to be involved in executive and cognitive behavioural functions [43,44,45]. Interestingly and based on neuropathological findings of several studies on autism post-mortem brains, cerebellum has been identified as one of the key brain regions that can play role in autistic features [46], and substantial accumulating evidence has linked the cerebellum with higher cognitive functions [47]. Moreover, the cerebellum is being considered a key structure within the social circuitry [48]. Furthermore, valproic acid as an environmental risk factor showed activation in different brain regions, with evidence of long-lasting glia activation in the hippocampus and the cerebellum [49], which are two brain regions linked to autism-related behaviour, namely social interaction and repetitive behaviours [46,47,48,49,50,51,52].

**Figure 1:**
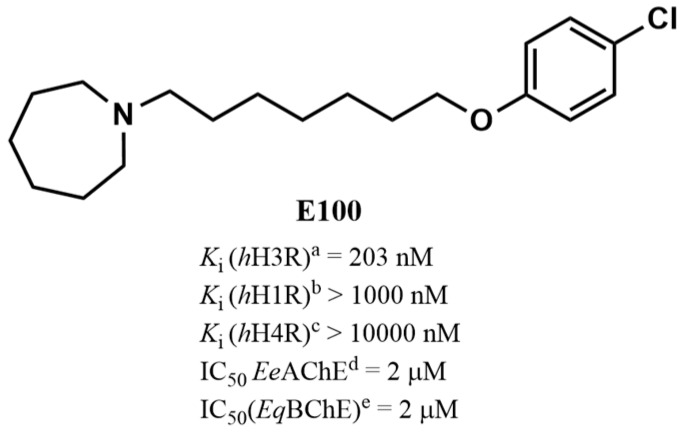
Chemical structure of the dual-acting human H3R (*h*H3R) antagonist and AChE inhibitor E100 and in vitro data with regard to *h*H1-, *h*H3-, and *h*H4R, *Ee*AChE, and *Eq*BuChE. ^a,b,c^ Binding assays to determine affinity to H1-, H3-, and H4Rs were performed in differently expressed cells as previously described n = 3 [59]. ^d^ AChE: Acetylcholine esterase; *Ee*; electric eel; ^e^ BuChE: Butyrylcholinesterase; *Eq*: equine [63,64,65].

Considering the aforementioned preclinical as well as clinical results, H3Rs represent a promising target for developing new dual-active compounds with the potential role in neuropsychiatric multi-neurotransmitter disorders, e.g., AD, cognitive deficit accompanying schizophrenia and ASD [1,29,53,54,55]. Given the involvement of AChE and H3-auto- and hetero-receptors in the modulation of several central neurotransmitters including ACh and HA, dual-active AChE inhibitors (AChEIs) and H3R antagonists have been developed by several groups [56,57,58,59,60,61,62]. Therefore, we describe the effects of a novel dual-active AChE inhibitor and H3R antagonist E100 (1-(7-(4-chlorophenoxy) heptyl) homo-piperidine) with balanced acetylcholine esterase inhibitory effect (*Ee*AChE: IC_50_ = 2 µM and *Eq*BuChE: IC_50_ = 2 µM), histamine H3 receptor (H3R) antagonist affinity (*h*H3R *K*_i_ = 203 nM), and high selectivity profile towards H3R subtype (Figure 1) in male C57BL/6 mice model of ASD, induced by prenatal exposure to VPA (500 mg/kg, i.p.). Moreover, the effects of E100 on locomotor activity and anxiety-like behaviors of the same animals were tested in the elevated plus-maze (EPM), since anxiety and motor activity can influence the performance of animals [62]. Furthermore, the effects of E100 on AChE activity and oxidative stress markers were assessed in the cerebellum, as it is involved in executive and cognitive functions and exaggerated oxidative stress may alter social behavior in adult mice [4,46]. In addition, the ability of the H1R antagonist mepyramine (MPA), H2R antagonist zolantidine (ZLT), H3R agonist (*R*)-α-methylhistamine (RAM), and cholinergic muscarinic antagonist scopolamine (SCO) to reverse the effects provided by E100 were evaluated to clarify whether brain HA and ACh are involved in the effects exhibited by E100.

## 2. Results

### 2.1. Effects of E100 on Sociability Impairments in Three-Chamber Behaviour (TCB) Test and Stereotyped Repetitive Behavior in Marble-Burying Behaviour (MBB)

**Figure 2:**
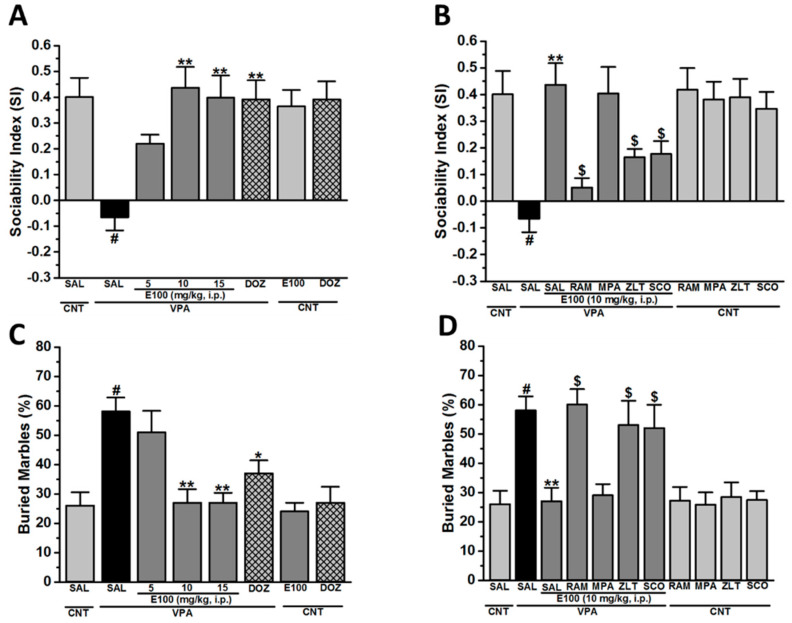
E100 improved sociability in three-chamber behaviour (TCB) and repetitive behavior in marble-burying behaviour (MBB) paradigms. (**A**,**B**) Following acclimatization for a duration of 10 min, male mice were allowed to explore all three chambers for 10 min. The obtained results were expressed in form of Sociability index (SI). Control (CNT) mice received systemic injections of saline (group 1), E100 (10 mg/kg) (group 7), and donepezil (DOZ) (1 mg/kg) (group 8), whereas VPA mice were injected with saline (group 2), E100 (5, 10, and 15 mg/kg) (groups 3–5), or DOZ (1 mg/kg, i.p.) (group 6) subchronically for 21 days (**A**). Abrogative studies of subchronic (21 days) systemic co-injection of RAM (10 mg/kg, i.p. for group 9), mepyramine (MPA) (10 mg/kg, i.p. group 10), zolantidine (ZLT) (10 mg/kg, i.p., for group 11), or scopolamine (SCO) (0.3 mg/kg, i.p., for group 12) on the E100 (10 mg)-provided improvement of sociability of VPA mice were assessed (**B**). Marble-burying behavior (MBB) was measured after a 30-min testing session applying the same treatments. VPA mice treated with saline (group 2) displayed significantly increased repetitive behaviors when compared to CNT mice (group 1). E100 (5, 10, or 15 mg/kg, i.p) or DOZ (1 mg/kg, i.p.) were injected systemically and subchronically for 21 days in VPA mice (**C**). Effects of subchronic (21 days) systemic co-administration of RAM (10 mg/kg, i.p., group 9), MPA (10 mg/kg, i.p., group 10), ZLT (10 mg/kg, i.p., group 11), or SCO (0.3 mg/kg, i.p., group 12) on the E100(10 mg)-provided attenuation of stereotyped repetitive behavior of VPA mice were assessed MBB (**D**). CNT mice were injected with saline, E100 (10 mg/kg, i.p.), DOZ (1 mg/kg, i.p.). RAM (10 mg/kg, i.p.), MPA (10 mg/kg, i.p.), ZLT (10 mg/kg, i.p.), or SCO (0.3 mg/kg, i.p.) (**D**). Data are expressed as the mean ± standard errors of the means (SEM) (n = 7 for TCB and n = 5 for MBB). 8 groups of 7 mice per group in TCB (**A**,**B**) and 8 groups of 5 mice per group in MBB (**C**,**D**) were used. The effects of E100 were analyzed using two-way analysis of variance (ANOVA) with dose of drugs and animals (either VPA or CNT mice) as the between-subjects factor, and post hoc comparisons were performed with Tukey’s test in case of a significant main effect. ^#^ *p* < 0.05 vs. CNT mice. ^**^ *p* < 0.01 vs. saline-treated VPA mice. ^$^ *p* < 0.05 vs. E100 (10mg)-treated VPA mice.

The effect of systemic injection of E100 (5, 10, and 15 mg/kg, i.p.) and donepezil (DOZ; 1 mg/kg, i.p.) on ASD-like sociability impairments in the three-chamber behaviour (TCB) task and stereotyped repetitive behavior in marble burying behavior (MBB) in VPA-exposed mice (VPA mice) are shown in Figure 2A,B. There was a statistically significant difference between groups as determined by statistical analyses (*F*_(7,48)_ = 5.118, *p* < 0.01). As observed in the Tukey post hoc analyses, VPA mice exhibited significantly lower sociability expressed as sociability index (SI) when compared to saline-exposed control mice (CNT), with SIs of (−0.07 ± 0.05) and (0.40 ± 0.07), respectively (*p* < 0.01) (Figure 2A). However, E100 (5, 10, and 15 mg/kg) significantly increased SI of VPA mice with SI values of (0.22 ± 0.08), (0.44 ± 0.08), and (0.40 ± 0.09) when compared to VPA mice with a SI of (−0.07 ± 0.05) (all *p* < 0.05) (Figure 2A). The results revealed that the enhancement in SI observed with E100 (10 mg/kg, SI = 0.44 ± 0.08) was statistically comparable to that shown with DOZ (1 mg/kg, SI = 0.39 ± 0.07, *p* = 0.99) (Figure 2A). Moreover, there was a statistically significant difference between tested groups in the abrogative study (*F*_(10,66)_ = 5.454, *p* < 0.01) (Figure 2B). Interestingly, the E100-provided improvement of sociability was counteracted following co-administration with RAM (10 mg/kg, i.p.), ZLT (10 mg/kg, i.p.), or SCO (0.3 mg/kg, i.p.), with SI values of (0.05 ± 0.04, *p* < 0.05), (0.16 ± 0.03, *p* < 0.05), and (0.18 ± 0.05, *p* < 0.05), respectively (Figure 2B). However, co-administration of the CNS-penetrant H1R antagonist MPA (10 mg/kg, i.p.) with SI value of (0.40 ± 0.10, *p* = 1.00) failed to reverse the E100 (10 mg/kg)-provided sociability enhancement observed in the E100 (10 mg/kg)-treated VPA animals (Figure 2B). Notably, subchronic treatment of CNT mice with E100 (10 mg/kg, i.p.), RAM (10 mg/kg, i.p.), MPA (10 mg/kg, i.p.), ZLT (10 mg/kg, i.p.), or SCO (0.3 mg/kg, i.p.) had no significant influence on SI compared to saline-pretreated CNT mice, with SI values of (0.36 ± 0.06, *p* = 1.00), (0.42 ± 0.08, *p* = 1.00), (0.38 ± 0.07, *p* = 1.00), (0.39 ± 0.07, *p* = 1.00), and (0.35 ± 0.06, *p* = 1.00), respectively (Figure 2B).

In the marble-burying behaviour (MBB) and as determined by statistical analyses, there was a significant difference between all groups assessed (*F*_(7,32)_ = 5.797, *p* < 0.01). Statistical post hoc analyses showed that VPA mice (58.00 ± 4.81%, *p* < 0.01) buried significantly more marbles compared to the CNT animals (26.00 ± 4.55%) tested in MBB (Figure 2C). However, E100 (10 or 15 mg/kg, i.p.) and DOZ (1 mg/kg, i.p.) significantly reduced the increased percentage of marbles buried by VPA mice when compared to saline-treated VPA mice, with (27.00 ± 4.60%, *p* < 0.01), (27.00 ± 3.34%, *p* < 0.01), and (37.00 ± 4.37%, *p* < 0.01), respectively (Figure 2C). Moreover, there was a statistically significant difference between tested groups in the abrogative study with (*F*_(10,44)_ = 5.935, *p* < 0.01) (Figure 2D). The E100 (10 mg)-provided a decrease in the percentage of buried marbles (27.00 ± 4.60%) was entirely abrogated by co-administration of RAM (60.00 ± 5.28%, *p* < 0.05), ZLT (53.00 ± 8.30%, *p* < 0.05), and SCO (52.00 ± 7.94%, *p* < 0.05), respectively (Figure 2D). However, MPA (29.00 ± 3.84%, *p* = 1.000) failed to counteract the E100 (10 mg)-provided effect (27.00 ± 4.60%) on VPA (Figure 2D). Notably, subchronic treatment of CNT mice with E100 (10 mg/kg, i.p.), RAM (10 mg/kg, i.p.), MPA (10 mg/kg, i.p.), ZLT (10 mg/kg, i.p.), or SCO (0.3 mg/kg, i.p.) had no significant influence on percentage of buried marbles compared to saline-pretreated CNT mice, with (24.00 ± 2.96%, *p* = 1.00), (27.20 ± 4.63%, *p* = 1.00), (25.80 ± 4.22%, *p* = 1.00), (28.40 ± 5.04%, *p* = 1.00), and (27.40 ± 3.00%, *p* = 1.00), respectively (Figure 2D).

### 2.2. Effects of E100 on Anxiety Levels and Locomotor Activity of Valproic Acid (VPA)-Exposed Mice in Elevated Plus Maze (EPM) Test

**Figure 3:**
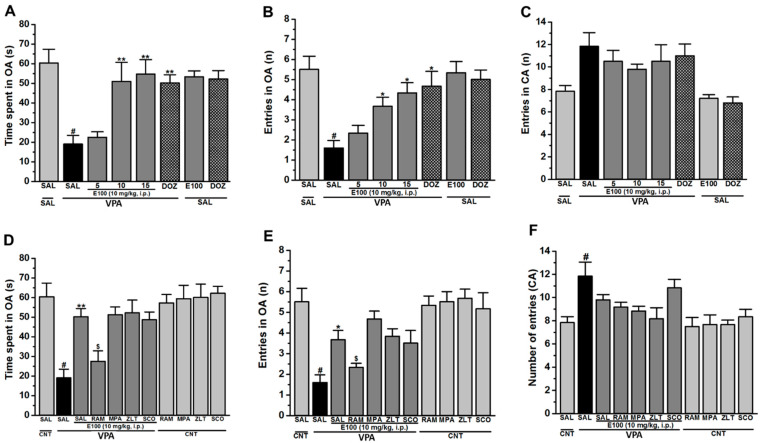
E100 ameliorated fear-related behavior without affecting locomotor activity in elevated plus maze (EPM). VPA mice injected with saline (group 2) displayed significantly increased deficits in cognitive behaviors compared to CNT mice (group 1). Test compound E100 (5, 10, or 15 mg/kg, i.p.) or DOZ (1 mg/kg) were injected for 21 days to VPA mice (subchronic). E100 (10 and 15 mg/kg, groups 4 and 5) and DOZ (1 mg/kg, group 6) attenuated the decreased time spent on the open arms, however, failed to modify the increased number of entries into the closed arms of the EPM (**A**–**C**). Abrogative effects of subchronic (21 days) systemic co-administration of RAM (10 mg/kg, group 9), MPA (10 mg/kg, group 10), ZLT (10 mg/kg, group 11), or SCO (0.3 mg/kg, group 12) on the E100 (10 mg)-provided improvement in number and time spent for open arms of VPA mice were measured (**D**,**E**). Number of entries into closed arms was elevated in saline-treated VPA mice (group 2) when compared to saline-treated CNT mice (group 1). E100 (5, 10, and 15 mg/kg) and DOZ failed to modulate the increased number of entries into closed arms (**C**,**F**). Additionally, CNT mice treated with E100 (10 mg/kg, group 7) did not show significant difference in number of entries into closed arms when compared with saline-treated CNT mice (group 1). Data are expressed as the mean ± SEM (n = 6). ^#^ *p* < 0.05 vs. CNT mice. * *p* < 0.05 vs. Saline-treated VPA mice. ** *p* < 0.01 vs. saline-treated VPA mice. ^$^ *p* < 0.05 vs. E100 (10mg)-treated VPA mice. In the EPM test, 8 groups of 6 mice per group were used. The effects of E100 were analyzed using two-way analysis of variance (ANOVA) with dose of drugs and animals (either VPA or CNT mice) as the between-subjects factor, and post hoc comparisons were performed with Tukey’s test in case of a significant main effect (**A**–**D**).

Figure 3A–F shows the observed effects of E100 (5, 10, or 15 mg/kg, i.p.) on the anxiety levels (the time spent (Figure 3A) and the number of entries into open arms (Figure 3B) of VPA mice assessed in the EPM test. Additionally, the locomotor activity expressed as the number of entries into closed arms (Figure 3C) was simultaneously evaluated in the same EPM test. Moreover, the abrogative effects of systemic co-administration of RAM, MPA, ZLT, or SCO on the E100-provided effects were tested (Figure 3D–F). The studies have been conducted for 5 min each. The results observed for the time spent and number of entries into open arms revealed a statistically significant difference between groups with (*F*_(10,55)_ = 5.505, *p* < 0.01) for time spent and (*F*_(10.55)_ = 7.339, *p* < 0.01) for number of entries (Figure 3A,B). Statistical post hoc analyses showed that VPA mice spent significantly less time (19.00 ± 4.46 s, *p* < 0.05) and displayed a lower number of entries (1.60 ± 0.37, *p* < 0.01) in open arms when compared to CNT mice with time spent (60.00 ± 7.02 s) and number of entries (5.50 ± 0.66) (Figure 3A,B). However, subsequent post hoc analyses revealed that E100 when administered at 10 or 15 mg/kg significantly altered the time spent exploring the open arms of the maze during a 5 min session compared to saline-treated VPA mice, with (50.17 ± 4.21 s, *p* < 0.05) and (54.67 ± 8.00 s, *p* < 0.05), respectively (Figure 3A). Moreover, post hoc evaluation revealed that E100 when administered at 10 or 15 mg/kg i.p. significantly increased the number of entries into the open arms of the maze during a 5 min session compared to saline-treated VPA mice, with (3.67 ± 0.54, *p* < 0.05) and (4.33 ± 0.51, *p* < 0.05), respectively (Figure 3B). However, E100 (5 mg/kg) failed to alter time spent and number for entries into open arms of VPA mice, with (20.98 ± 3.00 s, *p* = 0.57) and (2.41 ± 0.37), respectively, and as compared with VPA mice with (19.00 ± 4.46 s) and (1.60 ± 0.37) for time spent and number of entries, respectively (Figure 3A,B). Interestingly, VPA mice pretreated with DOZ (1 mg) spent significantly longer time exploring the open arms compared to saline-treated VPA mice, with (47.17 ± 4.46 s, *p* < 0.01) (Figure 3A). Further analyses of data describing the number of entries into the open arms of the maze yielded practically the same results for DOZ (1 mg/kg, i.p.), with (4.67 ± 0.73, *p* < 0.01) (Figure 3B). On the other hand, VPA mice entered the closed arms significantly more often than CNT mice, with (11.83 ± 1.22, *p* < 0.01) (Figure 3C). Besides, subchronic treatment of CNT mice with E100 (10 mg/kg, i.p.) or DOZ (1 mg/kg, i.p.) had no significant influence on time spent in open arms (Figure 3A), number of entries into open (Figure 3B) or into closed arms (Figure 3C) compared to saline-pretreated CNT mice (all *p* > 0.05). Interestingly, the E100 (10 mg)-provided increase in the time spent in open arms (Figure 3D), and the number of entries into open arms (Figure 3E) was entirely abrogated by co-administration of RAM (*p* < 0.05). However, MPA, ZLT, and SCO failed to counteract the E100 (10 mg)-provided effect on VPA mice (Figure 3D,E). Notably, the number of closed arm entries following subchronic systemic injection of E100 (5, 10, or 15 mg/kg) and DOZ (1 mg/kg, i.p.) was not significantly different as compared to saline-treated VPA mice, with (10.50 ± 0.75, *p* = 0.99), (9.50 ± 0.81, *p* = 0.88), (10.50 ± 1.45, *p* = 0.99), and (11.33 ± 1.24, *p* = 1.00), respectively (Figure 3C,F).

### 2.3. Effect of E100 on Oxidative Stress Levels in Cerebellar Tissues of VPA-Exposed Mice

**Figure 4:**
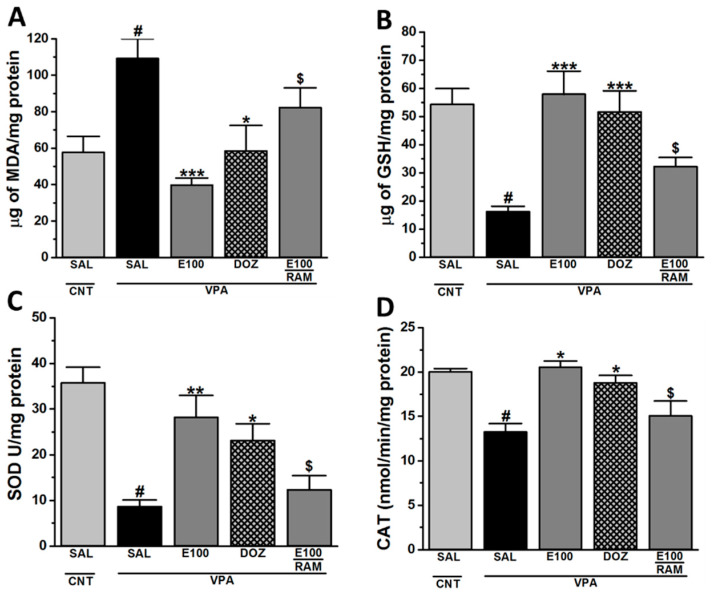
E100 restored levels of oxidative stress markers in the cerebellum. Modulated malondialdehyde (MDA), glutathione (GSH), catalase (CAT), and superoxide dismutase (SOD) were assessed. VPA mice showed a significant increase in MDA (**A**) and significant decrease in GSH (**B**), SOD (**C**), and CAT (**D**) compared to CNT mice. Subchronic systemic administration of E100 (10 mg/kg) or DOZ (1 mg/kg) were assessed in VPA mice. E100 (10 mg/kg) or DOZ (1 mg/kg) significantly reduced the increased levels of MDA (**A**) and significantly increased the reduced levels of GSH, SOD and CAT (**A**–**D**). Abrogative effects of subchronic (21 days) systemic co-administration with RAM (10 mg/kg) on modulation of oxidative stress levels provided by E100 (10 mg) were assessed (**A**–**D**). Data are expressed as the mean ± SEM (n = 5). ^#^ *p* < 0.05 vs. VPA mice. * *p* < 0.05 vs. VPA mice. ** *p* < 0.01 vs. VPA mice. *** *p* < 0.001 vs. VPA mice. ^$^ *p* < 0.01 vs. E100 (10 mg)-treated VPA mice. In biochemical assessments, 5 groups of 5 mice per group were used. The effects of E100 were analyzed using two-way analysis of variance (ANOVA) with dose of drugs and animals (either VPA or CNT mice) as the between-subjects factor, and post hoc comparisons were performed with Tukey’s test in case of a significant main effect (**A**–**D**).

As determined by statistical analysis, there was a significant difference between all groups assessed on the levels of MDA (malondialdehyde, *F*_(4.20)_ = 5.505, *p* < 0.01), GSH (glutathione, *F*_(4,20)_ = 7.399, *p* < 0.01), SOD (superoxide dismutase, *F*_(4,20)_ = 8.586, *p* < 0.01), and CAT (catalase, *F*_(4,20)_ = 8.057, *p* < 0.01) of VPA mice and following subchronic systemic administration of saline, E100 (10 mg/kg, i.p.), or DOZ (1 mg/kg, i.p.) (Figure 4A–D). The results showed that MDA was significantly increased (109.14 ± 10.84 µg/mg protein, *p* < 0.05) and GSH (16.16 ± 1.19 µg/mg protein, *p* < 0.05), SOD (8.66 ± 1.43 U/mg protein, *p* < 0.05), and CAT (13.24 ± 0.96 nmol/min/mg protein, *p* < 0.05) were significantly reduced in the cerebellum of VPA mice compared to CNT mice, with (57.66 ± 8.79 µg/mg protein) for MDA, (54.30 ± 5.59 µg/mg protein) for GSH, (35.72 ± 3.44 U/mg protein) for SOD, and (20.01 ± 0.37 nmol/min/mg protein) for CAT (Figure 4A–D). However, cerebellum of VPA mice pretreated with E100 (10 mg/kg, i.p.) displayed a significant reduction of MDA (39.69 ± 3.89 µg/mg protein, *p* < 0.001) as well as significant elevation of GSH (57.88 ± 8.23 µg/mg protein, *p* < 0.001), SOD (28.15 ± 4.82 U/mg protein, *p* < 0.01), and CAT (20.51 ± 0.73 nmol/min/mg protein, *p* < 0.05), and as compared with saline-treated VPA mice (Figure 4A–D). Similarly, cerebellum of VPA mice pretreated with DOZ (1 mg/kg, i.p.) showed a significant reduction of MDA (58.43 ± 14.00 µg/mg protein, *p* < 0.05) as well as significant increase of GSH (51.59 ± 7.51 µg/mg protein, *p* < 0.001), SOD (23.12 ± 3.66 U/mg protein, *p* < 0.05), and CAT (18.77 ± 0.84 nmol/min/mg protein, *p* < 0.05), and as compared with saline-treated VPA mice (Figure 4A–D). Moreover, systemic co-administration of RAM (10 mg/kg, i.p.) partially abrogated the E100 (10 mg)-provided decrease against the VPA-induced increase in the level of MDA (82.26 ± 10.80 µg/mg protein, *p* = 0.01) (Figure 4A), and it also partially reversed the E100 (10 mg)-provided increases in GSH (32.18 ± 3.22 µg/mg protein, *p* = 0.03), SOD concentrations (12.32 ± 3.08 U/mg protein, *p* = 0.04), and CAT (15.05 ± 1.68 nmol/min/mg protein, *p* = 0.02) in VPA mice (Figure 4B–D).

### 2.4. Effect of E100 on Acetylcholine Esterase Activity in Cerebellum Tissues of VPA-Exposed Mice

**Figure 5:**
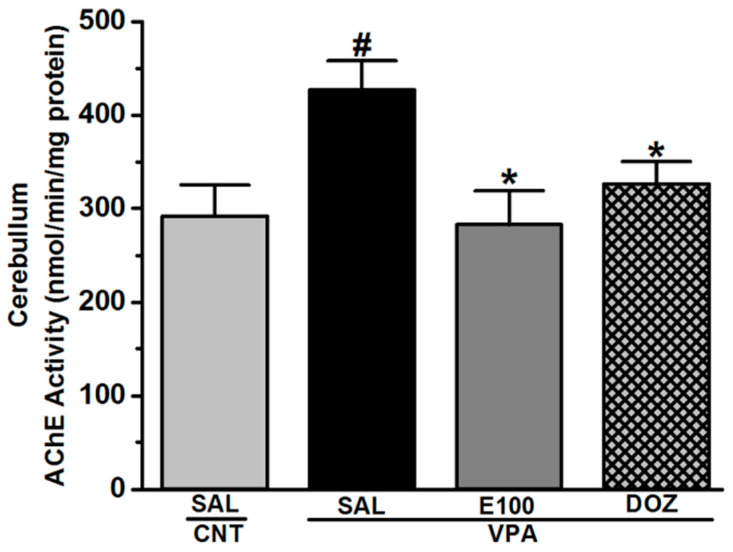
Effects of E100 on acetylcholine esterase activity in cerebellum tissues of valproic acid (VPA)-exposed mice. Inhibitory effects of E100 (10 mg/kg, i.p.) on acetylcholine esterase enzyme in the cerebellum of VPA mice. Quantitative analysis revealed a significant increase (^#^ *p* < 0.05) in the acetylcholine esterase enzyme activity in cerebellum of VPA mice compared to the CNT mice. However, subchronic treatment with E100 (10 mg/kg, i.p.) or DOZ (1 mg/kg, i.p.) to the VPA mice significantly reduced (* *p* < 0.05) this activity compared to the VPA mice. Values are expressed as the percent mean ± SEM. For assessment of AChE activity 4 groups were used. 4 CNT mice were used for saline group and 5 VPA mice were used for each treatment group. The effects of E100 were analyzed using two-way analysis of variance (ANOVA) with dose of drugs and animals (either VPA or CNT mice) as the between-subjects factor, and post hoc comparisons were performed with Tukey’s test in case of a significant main effect.

As determined by statistical analyses, there was a significant difference between groups assessed on the levels of AChE (*F*_(3,15)_ = 4.176, *p* < 0.05) (Figure 5). The observed results showed that VPA mice exhibited a significant increase in the activity of AChE enzyme in cerebellum (426.72 ± 31.20 nmol/min/mg protein, *p* < 0.05) when compared to CNT mice (291.87 ± 33.34 nmol/min/mg protein) (Figure 5). However, subchronic systemic treatment of VPA mice with E100 (10 mg/kg, i.p.) significantly decreased the AChE activity of VPA mice (282.87 ± 36.13 nmol/min/mg protein, *p* < 0.05) when compared with the saline-treated VPA mice (426.72 ± 31.20 nmol/min/mg protein) (Figure 5). Similarly, subchronic systemic pretreatment with the reference drug DOZ (1 mg/kg, i.p.) significantly decreased the AChE activity of VPA mice (326.16 ± 24.09 nmol/min/mg protein, *p* < 0.05) when compared with the saline-treated VPA mice (426.72 ± 31.20 nmol/min/mg protein) (Figure 5).

## 3. Discussion

Alteration in brain histaminergic and cholinergic neurotransmissions is supposed play a critical role in the clinical results of ASD-related behavioural features [1,25,66]. Consequently, the aim of the current study was to assess the modulating effects of the novel dual-acting AChEI and H3R antagonist E100 on brain HA and ACh applying a mouse model VPA-induced ASD-like behaviors. Our findings show that E100 significantly and dose-dependently improved sociability deficits in TCB paradigm and stereotypies in MBB paradigm in VPA mice. In the TCB paradigm, systemic pretreatment with E100 ameliorated the impairment in sociability demonstrated by VPA mice, since these animals, when pretreated with E100, showed significantly higher SI, with observed levels similar to the CNT mice. Various previous studies have focused on the procognitive effects of several H3R antagonists on social memory [67,68,69,70], a behavioral feature that is also altered in ASD [69]. Moreover, previous reports suggested that an impaired cholinergic system causes cognitive problems that may include social problems, which were reversed by donepezil treatments [71,72]. Importantly, the sociability-enhancing effect observed for E100 was dose-dependent, since E100 (10 mg/kg) showed an optimum effect comparable to that provided by the reference drug DOZ. Contrarily, a dose of 15 mg/kg E100 did not further improve upon the E100 (10mg)-provided sociability enhancement. The observations for the dose-dependent effects of E100 are in line with our recent studies observed with a non-imidazole-based H3R antagonist on VPA-induced ASD in Tuck-Ordinary mice [73], and an imidazole-based H3R antagonist in preclinical experiments in different rodents [1]. Moreover, the observations of sociability-enhancing effects for E100 aligns with earlier experimental results observed with the imidazole based H3R antagonist ciproxifan in Swiss mice [1], and comprehend our previously observations in which E100 enhanced social novelty in mice [63]. The mechanism behind E100-provided sociability improvement is unclear. Still, it might be explained by its dual action with the capability of H3R antagonists to mediate the release of different neurotransmitters other than histamine, such as DA, 5-HT, and ACh, in specific brain regions [74], together with its AChE inhibitory property that results in correction of abnormal cholinergic transmission. Interestingly, the E100-provided enhancing effects on sociability were reversed when mice were co-administered the H3R agonist RAM, the H2R antagonist ZLT, or with muscarinic cholinergic antagonist SCO, but not with the centrally acting H1R antagonist MPA, indicating that HA and ACh, through activation of postsynaptically located H2Rs (but not H1Rs) and muscarinic cholinergic receptors, respectively, obviously contribute to neuronal pathways important for alteration of sociability processes in the TCB paradigm in VPAmice. Therefore, considering the levels of different brain neurotransmitters, including HA and ACh, in various brain areas of the VPA-exposed mice with ASD-like behaviors as well as when pretreated with E100 would further assist in understanding the neural intersections involved in the observed behavioral enhancement. This is in vivo evidence that a simultaneous interaction with the above two targets leads to symptomatic in vivo enhancements of behavioral autistic-like parameters in mice. However, the mitigating effects observed for the dual-acting compound E100 are not due to either its AChE-inhibiting or to its H3R-blocking properties alone, since it neither acts purely as an H3R antagonist DL77 (non-imidazole based H3R antagonist [73]) or ciproxifan (an imidazole-based H3R antagonist [1]) nor as an AChEI such as DOZ (used as a reference drug in the current study). Notably, the postulated advantage of a dual-acting compound (e.g., E100) with combined affinities at the required targets over co-administration of two drugs are the straightforward single-compound pharmacokinetics. Thereby, putative drug–drug interactions occurring with combination therapy might be avoided. Conclusively, the dose-finding for co-application of two different drugs can be bypassed which would become necessary since the effective doses might be considerably different from the ones applied in the case of monotherapy, especially in multifactorial disorders like ASD. Whether the above mitigation of autistic-like behaviors is also induced after administration of H3R antagonist or co-administration of an H3R antagonist and an AChEI was beyond the scope of this project and will require dose-finding experiments for several ratios of the combination of AChEIs and H3R antagonist.

Repetitive behavior and restricted interests are considered as core features of patients with ASD [75,76]. In previous preclinical studies, abnormalities in histaminergic signalling was found to contribute to rare diseases such as Tourette syndrome [77], a condition featured by stereotypies and described to be among the most commonly comorbid neurodevelopmental disorders with ASD [1,63,73]. The results observed in the present study showed that VPA mice pretreated with E100 (10 or 15 mg) or with the reference drug DOZ (1 mg) demonstrated similar decreases in repetitive behavior when tested in MBB test, and the E100 (10 mg)-provided effects in MBB were nullified when mice were co-administered with RAM, ZLT, or SCO, but not with MPA. The mechanism by which the repetitive/compulsive behavior is improved following systemic administration with E100 could be explained with the capability of E100 to modulate the brain levels of different neurotransmitters in several specific brain areas besides HA and ACh, such as DA and 5-HT, through antagonist interaction of E100 with histamine H_3_ heteroreceptors expressed on dopaminergic and serotonergic neurons [78,79,80]. Furthermore, the results observed for E100 in MBB mirror a previous study in which acute systemic administration of the non-imidazole H3R antagonist DL77 significantly decreased the number of buried marbles in adult male Tuck-Ordinary mice of VPA-induced ASD features [73]. Interestingly, the results observed for E100 on repetitive behavior in MBB comprehend our previously observations for E100 in nestlet shredding behavioral test, a test that also evaluates repetitive/obsessive compulsive paradigm in rodents [63].

The effects of systemic administration with E100 on locomotion as well as anxiety levels were tested as pharmacological compounds that are able to modulate anxiety levels or locomotor activity may give rise to a false-positive effect in these behavioral paradigms. Therefore, locomotor activity was assessed simultaneously to exclude possible intrinsic deficits of spontaneous locomotor activity. Consequently, the numbers of entries into the closed arms were used as indicators of locomotor activity, while time spent and number of entries into open arms provided indications about anxiety levels in the EPM test. The results showed that E100 (10 or 15 mg) reduced elevated anxiety levels in VPA mice, similarly as DOZ (1 mg), measured by the time spent in open arms and number of open arms entries. However, pretreatment of VPA mice with E100 (5, 10, or 15 mg) or DOZ (1 mg/kg) did not alter a locomotor activity as measured by number of entries into closed arms. Moreover, subchronic co-administration of VPA mice with the most promising dose E100 (10 mg/kg, i.p.) and RAM, MPA, ZLT, or SCO had no significant influence on the number of closed arm entries compared to saline-pretreated CNT mice. Furthermore, subchronic systemic injection of CNT mice with the most promising dose E100 (10 mg/kg, i.p.) failed to modify locomotor activity of CNT mice in EPM test. These results comprehend our previously observations for E100 in open field assessment [63]. Thus, the improvements in sociability and repetitive/compulsive behaviors observed for E100 in TCB and MBB, respectively, appear unlikely to be associated with a modulating effect in locomotor activity of the tested mice. Moreover, the E100 (10 mg)-provided effects on anxiety-like behaviors of treated mice were nullified when mice were co-administered with RAM, but not with MPA, ZLT, or SCO, indicating that postsynaptic histaminergic receptor subtypes (H1Rs and H2Rs) and postsynaptic muscarinic cholinergic receptors are not involved in the E100-provided effects on anxiety-like behavior of VPA mice. However, the H3R agonist RAM abrogated the effects provided by E100, demonstrating that E100 may exerted its effects on anxiety-like behaviors VPA mice through modulation of several other neurotransmitters, such as serotonin [80,81], glutamate, and GABA [82,83,84], that are reported to be imbalanced in ASD patients. These results were in accordance with previous results that revealed anxiolytic-like effects of a non-imidazole-based H3R antagonist as well as UW-MD-71 with no differences in spontaneous locomotor activity [56,57,85].

Previous studies revealed that imidazole-based H3R antagonists, namely clobenpropit and ciproxifan, mitigated several oxidative stress markers (e.g., MDA and GSH) in amphetamine- or dizocilpine-augmented oxidative stress in a preclinical mice model of schizophrenia, signifying the protective effects of H3R antagonists in such conditions [86,87]. In the current study, the results showed that VPA mice with ASD-like behavioral features displayed significant increase in MDA, with a concomitant decline in GSH, SOD, and CAT in the cerebellum tissues, and several previous studies showed that cerebellum is significantly involved in executive and cognitive functions [39,42,43,83]. The observed results for E100 (10 mg/kg) and DOZ (1 mg/kg) showed a significant reduction of MDA as well as a significant elevation of GSH, SOD, and CAT. Moreover, systemic co-administration with RAM (10 mg) reversed the E100 (10 mg)-provided modulating effects on MDA, GSH, SOD, and CAT in VPA mice. The latter results indicate that modulation of brain histamine provided by E100 may have contributed to the correction of an unbalanced ratio of radical oxygen species through the generation of endogenous cellular antioxidant defensive mechanisms.

The ability of E100 to enhance cholinergic activity in VPA mice and to exert its potential effect on cognitive deficit associated with sociability impairments was confirmed by measuring AChE activity in the cerebellum. The results revealed that the activity of AChE in E100 (10 mg)-treated mice was significantly reduced compared to VPA mice, and comparable to the DOZ. Considering the role of this enzyme which is responsible in degrading the ACh, it has been reported in a previous study that AChE inhibition augmented ACh in the synapse, therefore, relieving the cognitive rigidity and ameliorating the social deficiency in VPA mice [25].

## 4. Materials and Methods

### 4.1. Animals

Bred in the local central animal facility of the College of Medicine and Health Sciences, United Arab Emirates University, C57BL/6 (C57) mice (aged 8–12 weeks, weighing 20–25 g) (Jackson Laboratory, Bar Harbor, ME, USA) were used in this study [88]. All mice were kept in plastic cages under a standard light/dark cycle, namely 12 h light cycle, and lights were switched on at 6:00 a.m. Additionally, animals were housed at constant temperature 22–25 °C, and with free access to tap water and a standard rodent chow diet. For mating, male and female mice were housed together, and female mice were observed daily. The day was considered as embryonic day 0 (E0) on which the vaginal plug was detected. Each pregnant female was then kept in a separate cage until delivery, the day of delivery was defined as postnatal day 0 (P0). The Institutional Animal Ethics Committee in the College of Medicine and Health Sciences/United Arab Emirates (Approval No. ERA-2017-5603) approved all procedures that were carried out in accordance with the recommendations of the European Communities Council Directive of 24 November 1986 (86/609/EEC).

### 4.2. Drugs

The dual-active AChE inhibitor and H3R antagonist E100, namely 1-(7-(4-chlorophenoxy)heptyl)azepane, was designed and synthesized in the Department of Technology and Biotechnology of Drugs Kraków, Poland, according to previously published procedures [65]. All chemical reagents used in the current study, including sodium valproate (VPA) (500 mg/kg, i.p.), donepezil hydrochloride (DOZ, 1 mg/kg, i.p.), the CNS-penetrant H3R agonist (*R*)-α-methylhistamine (RAM, 10 mg/kg, i.p.), the CNS-penetrant H1R antagonist pyrilamine (MPA) (10 mg/kg, i.p.), the CNS-penetrant H2R antagonist zolantadine (ZLT) (10 mg/kg, i.p.), and Scopolamine hydrochloride (SCO) (0.3 mg/kg, i.p.) were obtained from Sigma-Aldrich (St. Louis, MO, USA).The assay kit for reduced glutathione (GSH, Assay Kit, Lot no: 095M4114V, Product code: 1002170877) was obtained from Sigma-Aldrich (St. Louis, MO, USA). The lipid peroxidation assay kit for estimation of malondialdehyde (MDA, Lot no: MDA-2409, Product code: NWK-MDA01) was purchased from North West Life Science (Vancouver, WA, USA). The assay kits for superoxide dismutase (SOD, Batch no: 0538703, Item: 706002) and catalase (CAT, Batch no: 0539007, Item: 707002) were purchased from Cayman chemical (Ann Arbor, MI, USA). Acetylcholinesterase activity colorimetric assay kit (Lot no: GR 3295454-2, Product: ab65345) was purchased from BioVision (Milpitas, CA, USA). All the reagents used in the study were of analytical grade and were dissolved in 1% aqueous Tween 20 solution (saline) and administered intraperitoneally (i.p.) at a volume of 10 mL/kg adjusted to body weight, and all doses are expressed in terms of the free base. All the reagents used in the experiments were of analytical grade.

### 4.3. Study Design and Treatments

#### 4.3.1. Prenatal Treatment

On E12.5, pregnant females were intraperitoneally (i.p.) injected with either VPA 500 mg/kg [89,90], or saline and returned to their home cages, as described previously [63,73,89,90]. After the injection of VPA few pregnant mice died, and some gave still birth or underwent desorption. From the successfully delivered pups, only male offspring were used in the study. Pups delivered from VPA-exposed mothers were considered as VPA mice and were used for the experiments when they reached age of 8 weeks. On the other hand, pups delivered from mothers exposed to saline were used as CNT mice. All obtained offspring were weaned and gender-grouped (5–6 mice/cage) at P21.

#### 4.3.2. Postnatal Treatments

**Figure 6:**
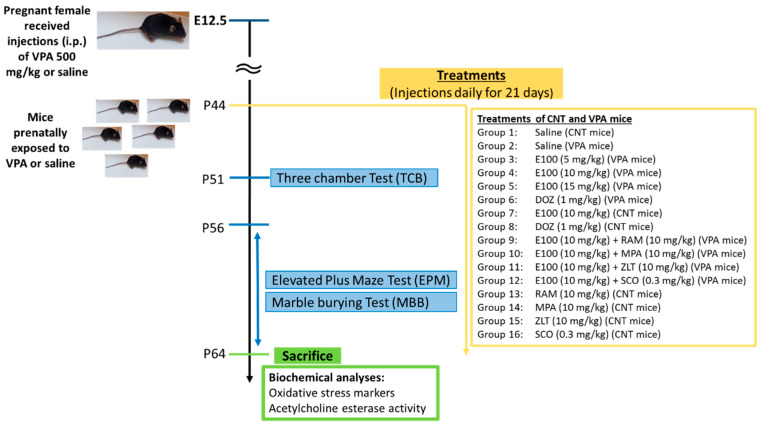
Schematic illustration of systemic treatments, behavioral experiments, and biochemical measurements with VPA and CNT mice. At embryonic day 12.5 (E12.5), pregnant mice were administered intraperitonially with VPA (500 mg/kg) or Saline. After delivery of pups and starting from postnatal day (P44), treatments of VPA mice and CNT mice were carried out. The systemic administrations continued for 21 days until VPA and CNT mice reached P64. Starting from P51, behavioral assessments were conducted. Following behavioral assessments, all mice were sacrificed at P64 for biochemical and immunofluorescence analyses. VPA and CNT treatment groups (8–12 mice/group) were subdivided into 16 subgroups and received intraperitoneally (i.p.) the following treatments and as shown in experimental design: VPA offspring (mice with autistic features; VPA) Group 1: CNT mice injected with saline, group 2: VPA mice injected with saline, group 3: VPA mice injected with E100 (5 mg/kg, i.p.), group 4: VPA mice injected with E100 (10 mg/kg, i.p.), group 5: VPA mice injected with E100 (15 mg/kg, i.p.), group 6: VPA mice injected with DOZ (1 mg/kg, i.p.), group 7: CNT mice injected with E100 (10 mg/kg, i.p.), group 8: CNT mice injected with DOZ (1 mg/kg, i.p.), group 9: E100 (10 mg/kg, i.p.) was co-administered with RAM (10 mg/kg, i.p.), group 10: E100 (10 mg/kg, i.p.) was co-administered with MPA (10 mg/kg, i.p.), group 11: E100 (10 mg/kg, i.p.) was co-administered with ZLT (10 mg/kg, i.p.), group 12: E100 (10 mg/kg, i.p.) was co-administered with SCO (0.3 mg/kg, i.p.), group 13: CNT mice injected with RAM (10 mg/kg), group 14: CNT mice injected with MPA (10 mg/kg, i.p.), group 15: CNT mice injected with ZLT (10 mg/kg, i.p.), and group 16: CNT mice injected with SCO (0.3 mg/kg, i.p.). All co-administrations were carried out in separate injections with 5-min interval following the test compound (E100) administration. CNT; control mice delivered from saline-exposed mice. VPA; autistic mice delivered from VPA-exposed mice.

On P21, male offspring (VPA mice with autistic features) from VPA-exposed mothers and from mothers that received saline (CNT, control mice) were divided into 16 subgroups (number of mice per group is provided in the following respective experimental sections), and received intraperitoneally (i.p.) the following treatment groups and as shown in experimental design (Figure 6): 1: CNT mice injected with saline, 2: VPA mice injected with saline (1% aqueous Tween 20), 3: VPA mice injected with E100 (5 mg/kg, i.p.), 4: VPA mice injected with E100 (10 mg/kg, i.p.), 5: VPA mice injected with E100 (15 mg/kg, i.p.), 6: VPA mice injected with DOZ (1 mg/kg, i.p.), 7: CNT mice injected with E100 (10 mg/kg, i.p.), 8: CNT mice injected with DOZ (1 mg/kg, i.p.), 9: E100 (10 mg/kg, i.p.) was co-administered with RAM (10 mg/kg, i.p.), 10: E100 (10 mg/kg, i.p.) was co-administered with MPA (10 mg/kg, i.p.), 11: E100 (10 mg/kg, i.p.) was co-administered with ZLT (10 mg/kg, i.p.), 12: E100 (10 mg/kg, i.p.) was co-administered with SCO (0.3 mg/kg, i.p.), 13: CNT mice injected with RAM (10 mg/kg), 14: CNT mice injected with MPA (10 mg/kg, i.p.), 15: CNT mice injected with ZLT (10 mg/kg, i.p.), and 16: CNT mice injected with RAM (10 mg/kg, i.p.). All co-administrations were carried out as separate injections with 5-min interval following administration of the test compound (E100). E100 (5, 10, and 15 mg/kg), or DOZ 1 mg/kg or vehicle (saline) were injected once daily for 21 days, from postnatal day (P44). All doses were selected based on the results of our previous studies of strongly related dual-active compounds and are expressed in terms of the free bases [57,85]. E100 and DOZ or saline were administered 30–45 min before each behavioral test, followed by a series of behavioral tests which began one week after starting the treatments and according to previously published works [63,73]. Doses for RAM, MPA, ZLT, and SCO were carefully selected according to previous experimental protocols from our laboratories. The behavioural experiments of the study were carried out between 9:00 a.m. and 3:00 p.m., and were conducted in the following sequence once the animals were 50 days old: three-chamber behaviour test (TCB), marble burying behaviour (MBB), and elevated plus maze (EPM). The behavioural assessments were carried out in the morning (8:00 a.m. and 12:00 p.m.) in a calm and sealed off area that was illuminated with four 60 V light-emitting diodes (LEDs). Before starting the behavioural tests, animals were habituated in the study place at least for one hour. To reduce the number of animals used, the levels of oxidative stress and AChE activity were studied in the same groups of animals that were subjected to behavioral tests.

### 4.4. Behavioral Tests

#### 4.4.1. Three-Chamber Behavior (TCB)

As previously described, the sociability test was performed [63,73,91,92]. It is a rectangular three chambered transparent polycarbonate cage (homemade), with one center chamber (40 cm × 20 cm × 22 cm) and two side chambers (40 cm × 20 cm × 22 cm) separated by two sliding doors. In the first session, a test mouse was habituated for 5 min in the center chamber with the two side doors closed. In the second session and following habituation, the doors were opened to allow the test mouse to explore all three chambers for a duration of 5 min. Before starting the third session, a stranger mouse of similar age, gender, and strain with no previous contact with the test mouse (referred to as a novel mouse (NM)), was positioned in a small plastic cage in the either left or right chamber, chosen randomly to avoid side preference, while the other cage was kept empty in the opposite chamber and was referred to as a novel object (NO). In the third session, the test mouse was allowed to explore all three chambers and cages for 10 min sociability test, and the time spent exploring the NM and NO (sniffing) was was automatically recorded during the experiment using EthoVision^®^ Software (Noldus, Netherlands). Finally, the time spent in the chamber with NM and around the cage was compared with the time spent in the chamber with NO. Eight groups of 7 mice/group were used for the TCB assessment. As previously described, sociability index (SI) was calculated by applying a mathematical equation to allow the direct comparison of social behavior of the treated groups [63,73], and was calculated with the following formula:

$$\mathrm{SI}\;=\;\frac{\mathrm{Time}\;\mathrm{exploring}\;\mathrm{novel}\;\mathrm{mouse}\;1\;-\;\mathrm{Time}\;\mathrm{exploring}\;\mathrm{novel}\;\mathrm{object}}{\mathrm{Time}\;\mathrm{exploring}\;\mathrm{novel}\;\mathrm{mouse}\;1\;+\;\mathrm{Time}\;\mathrm{exploring}\;\mathrm{novel}\;\mathrm{object}}.$$

#### 4.4.2. Marble Burying Behavior (MBB)

The test was performed as previously reported with slight modifications [63,93,94,95,96]. Briefly, each mouse was individually kept in a polycarbonate cage (26 cm × 48 cm × 20 cm) with fitted filter-top covers, and filled with fresh, unscented mouse bedding material to a depth of 5 cm, for habituation. After habituation for 10 min, the mouse was removed, and 20 glass marbles (15 mm diameter) were carefully overlaid equidistantly in a 4 × 5 arrangement in the cage. Each mouse was returned to its designated test cage and was allowed to explore for a duration of 30 min. The percentage of marbles buried (a marble was considered to be covered when >50% was covered by the bedding) was recorded and calculated as previously described [63]. Eight groups of 5 mice/group were used for the MBB assessment.

#### 4.4.3. Elevated Plus Maze (EPM) Test

The EPM test was performed as previously described with slight modifications [97,98,99,100]. Briefly, the maze is composed of two opposite open arms (30 cm × 6 cm), two opposite closed arms (30 cm × 6 cm × 15 cm) and a central area (6 cm × 6 cm) constructed from plexiglas. An animal was placed in the center of the maze facing an open arm. Mice entries as well as time spent into each arm were measured for 5 min using EthoVision^®^ Software (Noldus, Netherlands). The maze was carefully cleaned using a, with alcohol dampened, tissue (70%, *v*/*v*) to eliminate the odor of the previously assessed mouse. Eight groups of 6 mice/group were used in the EPM test.

### 4.5. Biochemical Assessments

#### 4.5.1. Brain Collection and Tissue Preparation for Biochemical Studies

Following behavioral assessments, the animals were sacrificed according to previously published protocols [63,73]. Deep anesthesia of the treated animals was achieved with pentobarbital (40 mg/kg, i.p.). Cardiac perfusion was carried out using 1× PBS (0.01 M phosphate buffer, 0.0027 M potassium chloride and 0.137 M sodium chloride) at pH 7.4 to wash out the blood. The perfusion was carried out manually by using a 50 mL syringe with 20 G needle. The optimal pressure was obtained by slowly flowing 1× PBS (approximately 5 mL/minute). The mice were observed until liver, heart, and kidney were blood free and gave a whitish color, an indication of blood removal. The brains were quickly removed and placed on an ice plate. The cerebellum was excised from the brain and snap-frozen in liquid nitrogen for further use in biochemical tests [63,73]. On the day of biochemical assessment, the tissues were homogenized and placed on ice in the extraction buffer recommended by the manufacturer, radioimmunoprecipitation assay (RIPA) buffer (50 mM Tris HCl, pH 7.4, 140 mM NaCl, 1 mM EDTA, 0.5% Triton X-100 and 0.5% sodium deoxycholate) )with protease and phosphatase inhibitors. The homogenates were sonicated and centrifuged for a duration of 30 min at 14,000 rpm and at 4 °C to eliminate tissue debris, and the resulting supernatant was used for the assessments of oxidative stress levels and AChE activity [88,89]. Five groups of 5 mice/group were used for oxidative stress marker estimations.

#### 4.5.2. Oxidative Stress Marker Estimations

##### Lipid Peroxidation Estimation

Malondialdehyde (MDA) detection kit was used to estimate the amount of lipid peroxidation after the manufacturer’s instructions, as previously described in our laboratories [63,101,102]. Briefly, samples or calibrators (250 μL) were incubated in the presence of acid reagent and thiobarbituric acid (250 μL). Then butylated hydroxytoluene in ethanol (10 μL) was added and vortexed vigorously. Samples were then incubated for 60 min at 60 °C and centrifuged at 10,000× *g* for 2–3 min. The reaction mixture was transferred to a cuvette aseptically and the absorbance was measured at 532 nm using VersaMax™ Microplate Reader (Molecular devices, San José, CA, USA). The ELISA reader was used from, tunable Microplate Reader with a SoftMax Pro reading software, wavelength range 340 nm to 850 nm. The protein estimation was performed by using BCA 96 well microplate method. Kits from Thermo Fisher Scientific (Waltham, MA, USA) (product number #23225) were obtained and the kits protocols were followed. Nunc MaxiSorp™ high protein-binding capacity 96 well ELISA plates were purchased from Thermo Fisher Scientific (product number #439454). The results are expressed as μM MDA/mg protein.

##### Glutathione (GSH) Estimation

The estimation of GSH levels were carried out according to the manufacturer’s instructions of the commercially available GSH kit purchased, and as reported earlier [63,101,102]. The reduced glutathione was estimated in the samples that were first deproteinized with 5% 5-sulfosalicylic acid solution and centrifuged to remove the precipitated protein. The obtained supernatant was used to assess the levels of GSH by measuring the absorbance of test samples at 412 nm with the kinetics for 5 min applying the microplate reader. The results are expressed as μM GSH/mg protein.

##### Estimation of Antioxidant Enzymes Activity

For estimation of the activity of antioxidant enzymes Superoxide dismutase (SOD) and Catalase (CAT), manufacturer’s instructions of commercially available kits were followed, and as previously reported [101,102]. CAT absorbance was read using a micro plate reader at 540 nm, and activity was expressed as nmol/min/mg protein. The protein estimation was carried out using the same devices and in similarity to the experimental protocol described under the Lipid Peroxidation Estimation Section. SOD absorbance was read at 450 nm using a microplate reader and activity was expressed as unit/mg protein.

##### Determination of Acetylcholinesterase (AChE) Activity in VPA-Exposed Mice Cerebellum

The acetylcholine assay kit was used, and the procedure followed was according to the manufacturer. The assay relates the hydrolysis of ACh to choline by AChE enzyme. Briefly, 5 µL of supernatant of homogenate (cerebellum tissue) was placed into the plate. Then, 45 µL of working reagent that consists of AChE assay buffer, and 50 µL reaction mix were added into each well. After incubation for 20–30 min at 37 °C, absorbance was read in a kinetic mode, and choosing two time points in a linear range to calculate the AChE activity of the sample, using VersaMax™ MicroplateReader with a tunnelable reader ranged from 340–850 nm wavelength.

### 4.6. Statistics

For behavioral studies and biochemical assessments, data were expressed as means ± SEM. The data were analyzed for normality by assessing the sample distribution or skewness (−1.8 to +1.8 considered normally distributed). After the results had passed the tests for normality, the effects of E100 were analyzed using two-way analysis of variance (ANOVA) with dose of drugs and animals (either VPA or CNT mice) as the between-subjects factor, and post hoc comparisons were performed with Tukey’s test in case of a significant main effect. For statistical comparisons, the software package SPSS 25.0 (IBM Middle East, Dubai, UAE) was used. The *p* values less than 0.05 were considered statistically significant.

## 5. Conclusions

**Figure 7:**
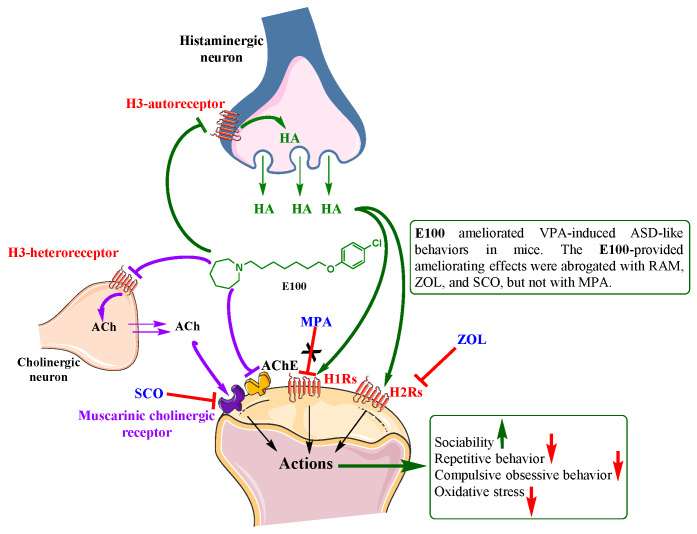
Putative mode of action of E100 by blocking the acting auto- and hetero histamine H3 receptors (H3Rs) and inhibition of the acetylcholine esterase enzyme (AChE). Regulating the release of brain histamine (HA) and acetylcholine (ACh), respectively and inhibiting the metabolism of ACh.

The novel dual-active H3R antagonist and AChE inhibitor E100 alleviated sociability deficits in TCB and stereotypies in MBB. In addition, E100 reduced elevated AChE activity and mitigated oxidative stress levels in cerebellum of mice with ASD-like behaviors induced by prenatal exposure to VPA (Figure 7). These results demonstrate the alleviating effects of E100 in different behavioural and biochemical assays following in vivo VPA-induced ASD in mice, and are to our knowledge the first in vivo demonstration that a potent dual-active H3R antagonist and AChE inhibitor is effective in improving sociability deficits and stereotypies of ASD-like features induced by prenatal exposure to VPA, and provide evidence to such dual-active compound to be used as a potential template for further drug design towards novel therapeutic entities for the treat ASD. However, additional trials with newer developed agents of this class and in different autistic animal models and in other species are warranted to clarify the pharmacological profile of the current class to develop proper, clinically potential candidates with balanced inhibitory affinities at both targets, namely the AChE and the H3Rs. Additionally, further investigations assessing pharmacokinetics/pharmacodynamics analysis for E100 are warranted to comprehend the provided ameliorating effects on ASD-like features and to exclude possible off-target effects.
